# Determination of Some Isoquinoline Alkaloids in Extracts Obtained from Selected Plants of the Ranunculaceae, Papaveraceae and Fumarioideae Families by Liquid Chromatography and In Vitro and In Vivo Investigations of Their Cytotoxic Activity

**DOI:** 10.3390/molecules28083503

**Published:** 2023-04-16

**Authors:** Justyna Misiurek, Tomasz Plech, Barbara Kaproń, Anna Makuch-Kocka, Małgorzata Szultka-Młyńska, Bogusław Buszewski, Anna Petruczynik

**Affiliations:** 1 Department of Inorganic Chemistry, Medical University of Lublin, Chodźki 4a, 20-093 Lublin, Poland; 2 Department of Pharmacology, Medical University of Lublin, Radziwiłłowska 11, 20-080 Lublin, Poland; tomasz.plech@umlub.pl (T.P.); anna.makuch-kocka@umlub.pl (A.M.-K.); 3 Department of Clinical Genetics, Medical University of Lublin, Radziwiłłowska 11, 20-080 Lublin, Poland; barbara.kapron@umlub.pl; 4 Department of Environmental Chemistry and Bioanalytics, Faculty of Chemistry, Nicolaus Copernicus University, Gagarina 7, 87-100 Torun, Poland; szultka.malgorzata@wp.pl (M.S.-M.); bbusz@chem.umk.pl (B.B.)

**Keywords:** cytotoxic activity, danio rerio larvae xenograft model, HPLC-DAD, LC-MS/MS, isoquinoline alkaloids, plant extracts

## Abstract

Alkaloids are heterocyclic bases with widespread occurrence in nature. Plants are rich and easily accessible sources of them. Most isoquinoline alkaloids have cytotoxic activity for different types of cancer, including malignant melanoma, the most aggressive type of skin cancer. The morbidity of melanoma has increased worldwide every year. For that reason, developing new candidates for anti–melanoma drugs is highly needed. The aim of this study was to investigate the alkaloid compositions of plant extracts obtained from *Macleaya cordata* root, stem and leaves, *Pseudofumaria lutea* root and herb, *Lamprocapnos spectabilis* root and herb, *Fumaria officinalis* whole plant, *Thalictrum foetidum* root and herb, and *Meconopsis cambrica* root and herb by HPLC-DAD and LC-MS/MS. For determination of cytotoxic properties, human malignant melanoma cell line A375, human Caucasian malignant melanoma cell line G-361, and human malignant melanoma cell line SK-MEL-3 were exposed in vitro to the tested plant extracts. Based on the in vitro experiments, *Lamprocapnos spectabilis* herb extract was selected for further, in vivo research. The toxicity of the extract obtained from *Lamprocapnos spectabilis* herb was tested using an animal zebrafish model in the fish embryo toxicity test (FET) for determination of the LC_50_ value and non-toxic doses. Determination of the influence of the investigated extract on the number of cancer cells in a living organism was performed using a zebrafish xenograft model. Determination of the contents of selected alkaloids in different plant extracts was performed using high performance liquid chromatography (HPLC) in a reverse-phase system (RP) on a Polar RP column with a mobile phase containing acetonitrile, water and ionic liquid. The presence of these alkaloids in plant extracts was confirmed by LC-MS/MS. Preliminary cytotoxic activity of all prepared plant extracts and selected alkaloid standards was examined using human skin cancer cell lines A375, G-361, and SK-MEL-3. The cytotoxicity of the investigated extract was determined in vitro by cell viability assays (MTT). For in vivo determination of investigated extract cytotoxicity, a *Danio rerio* larvae xenograft model was used. All investigated plant extracts in in vitro experiments exhibited high cytotoxic activity against the tested cancer cell lines. The results obtained using the *Danio rerio* larvae xenograft model confirmed the anticancer activity of the extract obtained from *Lamprocapnos spectabilis* herb. The conducted research provides a basis for future investigations of these plant extracts for potential use in the treatment of malignant melanoma.

## 1. Introduction

Malignant melanoma is one of the most aggressive skin cancers, and its incidence and mortality are increasing worldwide [1]. In the 27 European Union countries (EU 27) in 2020, this type of cancer accounted for 4% of all cancer diagnoses and for 1.3% of all deaths due to cancer (European Cancer Information System ECIS) [2].

Numerous alkaloids have in vitro and in vivo anticancer activity against various types of cancers. The alkaloids vinblastine, vincristine, vinorelbine and vindesine have been used in cancer treatment for many years [3]. Further studies on the potential anticancer activity of plant-derived compounds such as alkaloids are highly needed; approximately 25% of all newly approved anti-cancer drugs are related to natural products [4]. Natural compounds are frequently less toxic, and they show fewer side effects. Their relatively low price and often high availability are also important.

*Macleaya cordata* is a plant growing wild in Shanxi, Guizhou and Yunnan provinces in China. It is widely used in traditional Chinese medicine for the treatment of injuries, arthritis, rheumatic arthralgia and trichomonas vaginalis [5]. In North America and Europe, *Macleaya cordata* is also a traditional medicinal plant used as a remedy for insect bites [6] and ringworm infection [7]. Extracts from *Macleaya cordata* and their components have many biological properties, such as anti-microbial [8] anti-fungal [9], pesticidal [10] and anticancer properties [11]. Current pharmacological studies have shown that *Macleaya cordata* stimulates the growth of animals [12]. *Macleaya cordata* contains many biologically active compounds, mostly the alkaloids sanguinarine, chelerythrine, protopine, and allocryptopine [13].

The small genus *Pseudofumaria Medik*. was separated from the genus *Corydalis* only on the basis of morphological features. *Pseudofumaria* consists of only two species: *Pseudofumaria lutea* (L.) *Borkh* (*syn. Corydalis lutea* (L.) *DC.*) and *Pseudofumaria alba* (*Mill.*) *Lidén* (*syn. Corydalis alba* (*Mill.*) *Mansf*) [14]. *Pseudofumaria lutea* grows in nature on shady limestone rocks and screes in the Italian and Swiss Alps. It is also cultivated as an ornamental plant throughout Europe, which has given it status as an anthropophyte [15,16]. *Pseudofumaria lutea* is a perennial with branched stems and many 2–3 pinnate leaves. The plant produces yellow flowers gathered in racemes. The plant flowers from May to October [17]. The major active constituents of *Pseudofumaria lutea* are isoquinoline alkaloids: coptisine, berberine, protopine, sanguinarine, allocryptopine, chelidonine and chelerythrine [18]. *Pseudofumaria lutea* extracts have a large range of pharmacological activities, including antibacterial, antiviral and anticancer properties [18,19,20]. 

*Lamprocapnos* is monotypic (*Lamprocapnos spectabilis* (Linnaeus) Fukuhara) (=*Dicentra spectabilis* (L.) Lem.) and is the sister group to the subfamily *Fumarioideae* [17,21]. The roots of *Lamprocapnos spectabilis* contain coptisine, cheilantifoline, scoulerine, and protoberberine. The plant has been used in traditional medicine for paralysis, strokes, bruises, blood circulation and anti-inflammation [22]. Earlier phytochemical studies on *Lamprocapnos spectabilis* reported the isolation of fungitoxic alkaloids [23] and compounds with apoptosis-inducing activities [24]. *Lamprocapnos spectabilis* has very high cytotoxic activity against MDA-MB-231, FaDu, MCF-7 and SCC-25 cancer cell lines [20].

*Fumaria officinalis* is a leafy plant that belongs to the *Fumariaceae* family [25]. *Fumaria officinalis* has varied usage in herbal medicine all over the world. The extract of this plant is used in traditional medicine to cure, among others, stomachache, rheumatism, and skin disorders [26,27]. *Fumaria officinalis* contains many groups of compounds, but the main components that have a therapeutic effect are alkaloids such as protopine, sanguinarine and fumaritine [28]. The antitumor, antimicrobial, antioxidant, antifungal, antiviral and antispasmodic effects of *Fumaria officinalis* extract have been reported [20,28,29,30,31].

The *Meconopsis* genus, known as the “Blue poppy”, belongs to the *Papaveraceae* family and contains over 70 species. The area of the eastern Himalayas and the Hengduan Mountains is the main habitat of the genus *Meconopsis* [32,33]. According to Tibetan ancient medicinal literature and Flora of Tibet, *Meconopsis* species might have the capability of clearing heat antioxidants, relieving cough and asthma, analgesia, and anti-inflammation, and protecting the liver [34]. Alkaloids and flavonoids are the main active constituents inducing the pharmacological responses. *Meconopsis cambrica* (L.) Vig. (*Papaveraceae*) is a perennial herb and the only European representative of an otherwise Himalayan genus of *Meconopsis*. As a natural plant, it is local to western Europe, growing in Spain, France, Ireland, southwest England and Wales [35].

Plants belonging to the genus *Thalictrum* (*Ranunculaceae*) contain different classes of isoquinoline alkaloids that exhibit anti-infectious, antitumor, anti-parasite and platelet aggregation effects [36]. *Thalictrum foetidum* is a high perennial stark herb local to China (Yunnan, Sichuan and Tibet) [37]. In traditional medicine, the extracts of its roots have been used to heal enteritis, sore throat, and dysentery [38]. Several investigations have been conducted on the cytotoxic activity of *Thalictrum foetidum* extracts [39]. *Thalictrum acutifolium* also showed apoptosis-inducing activity for the human non-small cell lung cancer (NSCLC) cell line PLA-801 [40] and a cultured, highly metastatic human lung cancer cell line, 95-D [41]. 

The aim of this study was the determination of the alkaloid contents in plant extracts obtained from *Macleaya cordata* root, stem and leaves, *Pseudofumaria lutea* root and herb, *Lamprocapnos spectabilis* root and herb, *Fumaria officinalis* whole plant, *Thalictrum foetidum* root and herb, and *Meconopsis cambrica* root and herb by HPLC-DAD. The presence of these alkaloids in the investigated plant extracts was confirmed by LC-MS/MS. We also in vitro investigated the cytotoxic activities of alkaloid standards and plant extracts. Further, in vivo antitumor effects of the extract obtained from the herb of *Lamprocapnos spectabilis* were tested using the zebrafish (*Danio rerio*) xenograft model. To the best of our knowledge, the cytotoxic activity of this plant extract has not been previously investigated in vivo. The obtained results indicated the need for further investigations on the potential use of the tested plant extracts for the treatment of melanoma.

## 2. Results and Discussion

### 2.1. HPLC-DAD Analysis of Alkaloid Standards and Plant Extracts

**Figure 1:**
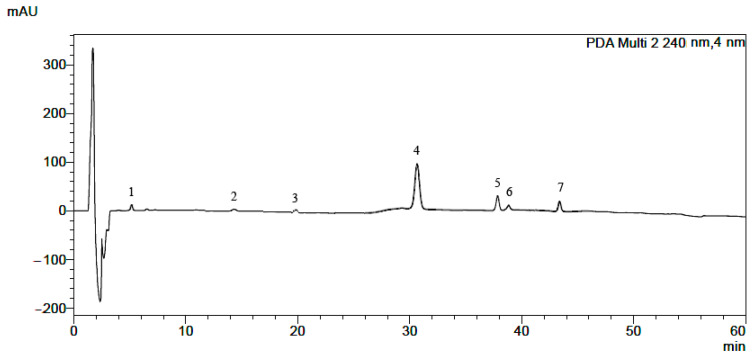
HPLC-DAD chromatogram obtained for mixture of alkaloid standards: 1—magnoflorine, 2—protopine, 3—stylopine, 4—palmatine, 5—berberine, 6—sanguinarine, 7—chelerythrine.

**Figure 2:**
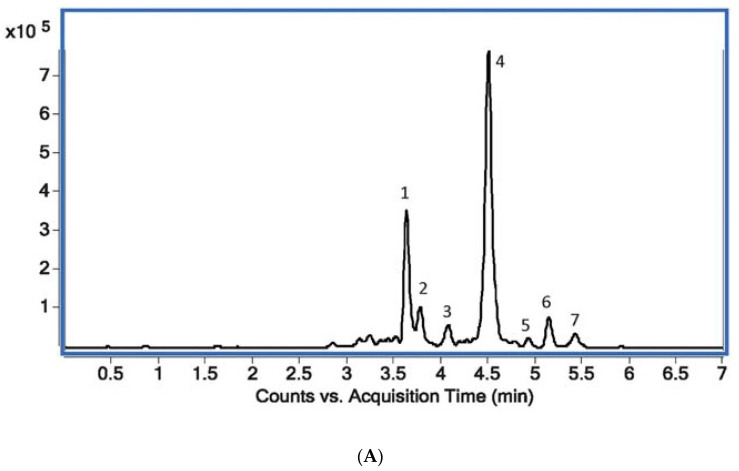

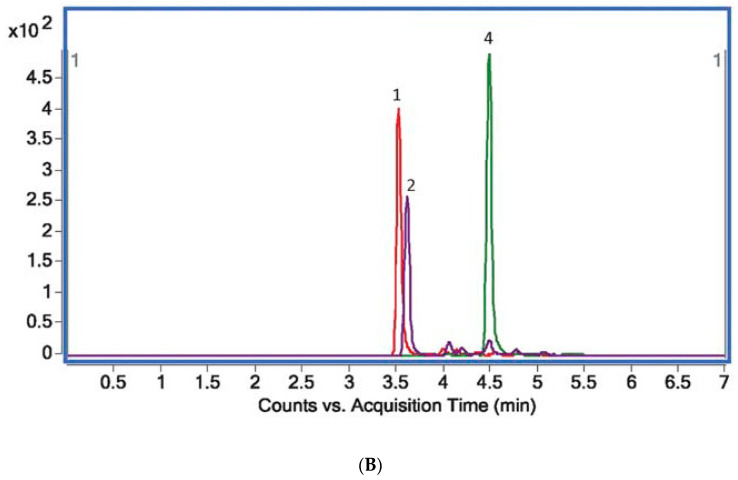
(**A**) Total ion MS chromatogram (TIC) showing standards of target alkaloids obtained with the use of UHPLC Q-TOF-MS: 1—protopine (*m*/*z* = 353.7655), 2—sanguinarine (*m*/*z* = 331.7065), 3—palmatine (*m*/*z* = 351.7853), 4—chelerythrine (*m*/*z* = 347.7489), 5—magnoflorine (*m*/*z* = 341.7917), 6—stylopine (*m*/*z* = 331.7105), 7—berberine (*m*/*z* = 335.7429). (**B**) An exemplary extracted ion chromatogram (EIC) for plant sample (*Macleaya cordata* leaves) (**B**).

The isoquinoline alkaloid standards berberine, chelerythrine, magnoflorine, palmatine, protopine and sanguinarinee (Table 1) were chromatographed on a Polar RP column with mobile phase containing acetonitrile, water and 0.04 ML^−1^ of 1-butyl-3 methylimidazolium tetrafluoroborate in a gradient elution system as described in the Experimental Section. The chromatographic conditions were based on a previously published procedure after appropriate modification [42]. The π–π interaction retention times (t_R_), asymmetry factors (As), and theoretical plate number per meter (N/m) obtained for the investigated alkaloid standards with the chromatographic system containing addition of ionic liquid in the mobile phase and phenyl stationary phase are presented in Table 1. The application of the chromatographic system with double protection against undesirable interactions of basic analytes with free silanol groups allowed us to obtain high system efficiency, symmetrical peaks, and full separation of the investigated alkaloids. For all alkaloids, As values between 0.99 and 1.11 and high N/m values (from 21,000 to 490,000) were obtained (Table 1). Typical chromatograms obtained by HPLC-DAD and LC-MS for mixtures of the investigated alkaloid standards are presented in Figure 1 and Figure 2.

**Figure 3:**
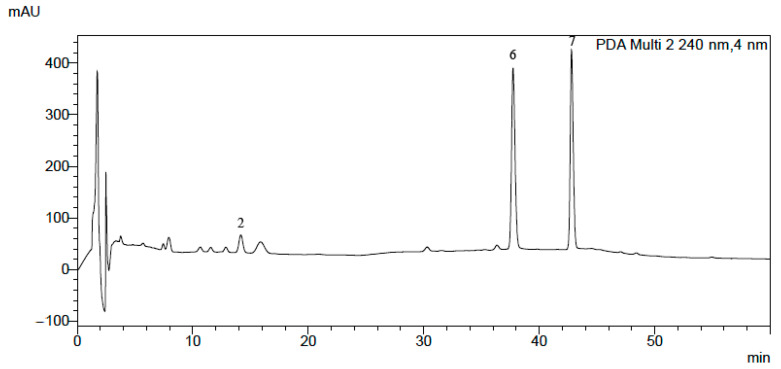
HPLC-DAD chromatogram obtained for *Macleaya cordata* leaves. For abbreviations, see Figure 1.

Using the same chromatographic system, we performed an analysis of the alkaloids in the plant extracts obtained from *Macleaya cordata* leaves, stalk and root, *Pseudofumaria lutea* herb and root, *Lamprocapnos spectabilis* herb and root, *Fumaria officinalis*, *Meconopsis cambrica* herb and root, and *Thalictrum foetidum* herb and root. An exemplary chromatogram obtained for *Lamprocapnos spectabilis* root extract is presented in Figure 3. For confirmation of the presence of alkaloids in the plant extracts, we used comparisons of their retention times with the retention times of alkaloid standards, UV-Vis spectra, and MS and MS/MS spectra (Figure S1). Detailed descriptions of the fragmentation patterns for each of the studied alkaloids were published previously [20,42].

Table 2 summarizes MS-based information (adduct forms, observed mass and product ions). Highly abundant protonated [M + H]+ ions in all of the studied alkaloids were observed in the ESI mass spectra due to the strong basicity of the secondary or tertiary amine groups. Hence, the relevant isoquinoline alkaloids were identified based on MS spectra for berberine (*m*/*z* = 335.7429), chelerythrine (*m*/*z* = 347.7489), stylopine (*m*/*z* = 331.7105), palmatine (*m*/*z* = 351.7853), magnoflorine (*m*/*z* = 341.7917), protopine (*m*/*z* = 353.7655) and sanguinarine (*m*/*z* = 331.7065). Moreover, their presence in different morphological parts of real plant samples was confirmed by MS/MS spectra and collision-induced dissociation (CID) of the peak of the most intense ion. Each time, the MS ions were detected with the use of a total ion chromatogram (TIC) mode. The optimization of different MS parameters for selectivity and MS response (in terms of the TIC peak areas) for the studied compounds was carried out without a chromatographic column. After determining the best conditions for isolating the precursor ion (analyte proton adduct), full-scan MS/MS mode was used to record produced ions from the real samples of each target compound. The fragmentation amplitude and isolation width for each analyte were manually optimized to increase the method’s selectivity and sensitivity and to select the most intense and characteristic fragmentation ions for qualitative analysis. The identified alkaloids were further characterized based on the MS/MS fragmentation patterns.

The quantitative analysis was performed by a calibration curve method. The number of replicates was three for all concentrations of all alkaloids. Calibration curve equations, correlation coefficients (r), limit of detection (LOD), and limit of quantification (LOQ) obtained for the alkaloids are presented in Table 3.

The results of the quantitative determination of alkaloids in the investigated plant extracts are presented in Table 3. Large differences in the content of alkaloids between the various plants and their individual parts were observed (Table 4). Magnoflorine was determined only in the extracts obtained from *Thalictrum foetidum*. The root of this plant contained 0.021 mg magnoflorine per 1 g of dry plant material. Protopine was identified in most of the investigated extracts. The highest amount of this alkaloid was determined in the extract obtained from the root of *Lamprocapnos spectabilis* (3.350 mg/g of dry plant material). Stylopine was found in *Pseudofumaria lutea* extracts. In the *Pseudofumaria lutea* herb and root extracts, 0.655 and 5.716 mg of stylopine per gram of dry plant material were determined, respectively. The root of the plant contained nearly nine times more stylopine than herb. Palmatine was identified in *Pseudofumaria lutea* root and herb. The highest content of this alkaloid was found in the extract obtained from *Pseudofumaria lutea* root (0.268 mg/g of dry plant material). Berberine was found in *Thalictrum foetidum* root (0.308 mg/g of dry plant material), and in a slight amount in *Thalictrum foetidum* herb (about 0.001 mg/g). Sanguinarine was determined in the extracts obtained from *Lamprocapnos spectabilis* herb and root, *Fumaria officinalis*, *Macleaya cordata* leaves, stalk and root, and *Meconopsis cambrica* herb and root. The content of this alkaloid ranged from 0.005 mg/g of dry plant material for the extract obtained from *Meconopsis cambrica* herb to 0.097 mg/g of plant material for the extract obtained from *Lamprocapnos spectabilis* root. Chelerythrine was identified in three investigated extracts obtained from the leaves, stalk and root of *Macleaya cordata*. The content of chelerythrine in the leaves (0.046 mg/g of dry plant material) was about two times higher than that in the stalk and root. For accuracy measurements, plant samples were spiked with alkaloids determined in these extracts. Recovery rates ranged from 87.5% to 104.5%.

### 2.2. Investigation of In Vitro Cytotoxic Activity of Plant Extracts

The cytotoxic activity of the investigated extracts obtained from *Macleaya cordata* root, stem and leaves, *Psudofumaria lutea* root and herb, *Lamprocapnos spectabilis* root and herb, *Fumaria officinalis* whole plant, *Thalictrum foetidum* root and herb, and *Meconopsis cambrica* root and herb was tested against three melanoma cell lines (A375, G-361 and SK-MEL-3). The obtained results were expressed as IC_50_ values (Table 5).

All investigated plant extracts exhibited cytotoxic activity against all tested cell lines. The extracts from *Macleaya cordata* leaves, stalk, and root strongly inhibited the viability of all tested melanoma cell lines. The obtained IC_50_ values were low (from 0.14 µg/mL to 7.13 µg/mL). The strongest cytotoxic activity was observed for the extracts obtained from stalk and leaves against SK-MEL-3 cells, with IC_50_ = 0.14 and 0.21 µg/mL, respectively. The cytotoxic activity of *Macleaya cordata* extracts against some cancer cell lines, e.g., adenocarcinoma epithelial cells (A549) was previously reported [11], but there are no reports on the cytotoxic activity of the extracts from this plant against melanoma cells. The cytotoxic activity of sanguinarine obtained from *Macleaya cordata* was also tested in vivo against human colon carcinoma cell lines (SW480) using a nude mouse xenograft model [43].

The extracts obtained from *Pseudofumaria lutea* root and herb showed the highest activity against G-361 cells (IC_50_ = 6.63 and 7.96 µg/mL, respectively). It was previously reported that extracts from this plant decreased the viability of human pharyngeal squamous carcinoma cells (FaDu), human tongue squamous carcinoma cells (SCC-25), the human breast adenocarcinoma cell line MCF-7, and human triple-negative breast adenocarcinoma cell line MDA-MB-231, with IC_50_ values ranging from 29.37 to 57.98 µg/mL [20].

*Lamprocapnos spectabilis* herb extract significantly inhibited viability of A375 cells with IC_50_ value of 4.13 µg/mL. In previous study extract obtained from herb of the plant showed cytotoxicity against FaDu, SCC-25, MCF-7 and MDA-MB-231 cell lines with IC_50_ values of 19.88, 29.55, 11.66 and 9.66 µg/mL, respectively [20]. Cytotoxic activity of *Lamprocapnos spectabilis* extracts have not been previously tested against melanoma cells.

The *Fumaria officinalis* extract showed the highest cytotoxicity against G-361 cells, with an IC_50_ value of 11.79 µg/mL and low cytotoxic activity against the other two tested cell lines. The extracts obtained from the plant slightly inhibited the viability of FaDu, SCC-25, MCF-7 and MDA-MB-231 (IC_50_ from 85.6 to >200 µg/mL) [20]. Nanoparticles with *Fumaria officinalis* also slightly inhibited the viability of human ovarian cancer cell lines: PA-1, Caov-3, SW-626, and SK-OV-3, with IC_50_ > 200 µg/mL [44].

The *Meconopsis cambrica* herb extract most strongly inhibited the viability of G-361 cells, with an IC_50_ value of 11.72 µg/mL. In a previous report, the highest cytotoxicity of the extract obtained from *Meconopsis cambrica* herb was observed against FaDu cells, with an IC_50_ value of 13.7 µg/mL [20].

The highest cytotoxic activity of the extracts obtained from *Thalictrum foetidum* root and herb was determined against G-361 cells, with IC_50_ values of 6.69 and 9.72 µg/mL, respectively. Significant cytotoxic properties of *Thalictrum foetidum* root extract against FaDu cells (IC_50_ = 4.98 µg/mL) were described previously [39].

The extracts obtained from *Macleaya cordata* leaves, stalk and root and *Lamprocapnos spectabilis* herb exhibited higher cytotoxic activity against A375 cells than cisplatin, an anticancer drug (IC_50_ = 23.8 µg/mL).

### 2.3. In Vivo Anticancer Activity of Lamprocapnos spectabilis Extract

#### 2.3.1. In Vivo Investigation of Toxicity of *Lamprocapnos spectabilis* Herb Extract to Determine LC50 Value and Non-Toxic Doses

**Figure 4:**
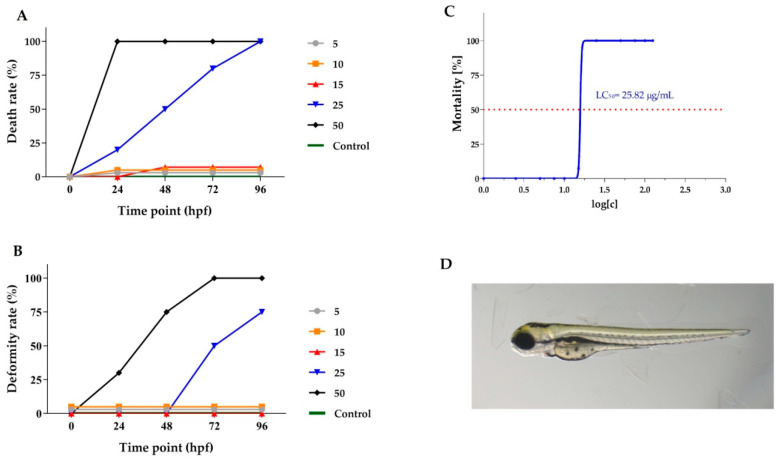
Toxicological analysis of *Lamprocapnos spectabilis* extract (concentration expressed in μg/mL). (**A**) Time–response curves of zebrafish embryo death rate during incubation with a dilution series of the *L. spectabilis* extract. (**B**) Time–response curves of deformity rate at tested doses. (**C**) Mortality of *D. rerio* larvae in a concentration-dependent manner to *Lamprocapnos spectabilis* extract exposure. The median lethal concentration (LC_50_) was based on cumulative mortality obtained from three independent experiments at 96 h post fertilization (hpf). (**D**) Representative pictures of 96 hpf zebrafish exposed to 10 μg/mL *Lamprocapnos spectabilis* extract.

The *Lamprocapnos spectabilis* herb extract was selected for further research using an animal model because the cytotoxic activity of the extract obtained from the plant had not been studied in vivo previously. The extract contained significant amounts of isoquinoline alkaloids and exhibited significant activity against the A375 cell line (IC_50_ of 4.13 µg/mL). Viability and malformation rates of *Danio rerio* larvae after exposure to different concentrations of *Lamprocapnos spectabilis* herb extract were determined at 24, 48, 72 and 96 hpf (Figure 4A,B). The exposure to *Lamprocapnos spectabilis* herb extract at concentrations of 25 and 50 μg/mL caused 100% mortality of the *Danio rerio* embryos and larvae at 96 hpf (Figure 4A). After exposure to lower concentrations of the investigated extract, mortality was compared to that of the control group. The embryos treated with 25 and 50 μg/mL of *Lamprocapnos spectabilis* herb extract showed malformations during this assay. Lower extract concentrations did not cause malformations (Figure 4B).

The median lethal concentration (LC_50_) based cumulative mortality obtained at 96 hpf was determined as 25.82 µg/mL (Figure 4C). A representative photo of *Danio rerio* larva exposed to 10 μg/mL *Lamprocapnos spectabilis* extract is presented in Figure 4D.

#### 2.3.2. *Danio rerio* Human Tumor Cell Xenograft

**Figure 5:**
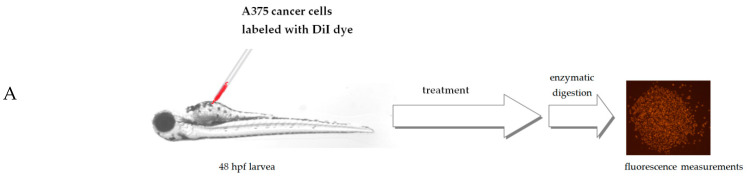

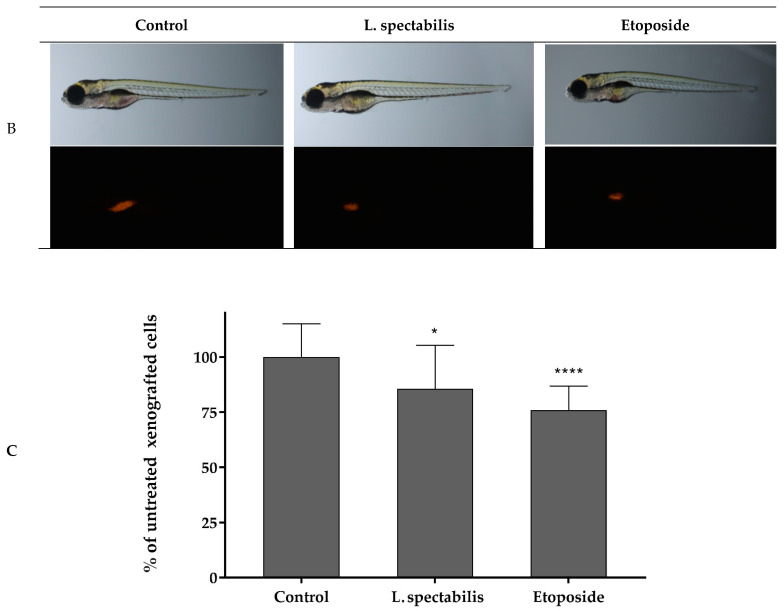
Antitumor activity of *Lamprocapnos spectabilis* extract in vivo. (**A**) Schematic diagram of the experiment based on the zebrafish human tumor xenograft model. (**B**) Images of 96 hpf (hours post fertilization)/48 hpi (hours post injection) larvae. Zebrafish larvae were xenografted with A375 cells (an average of 500 cells) and treated with 7.5 μg/mL of *Lamprocapnos spectabilis* extract or 5 μg/mL of a reference drug—etoposide or fish medium E3—as a control (10 animals/group). (**C**) Inhibition of cancer cell proliferation in vivo. * *p* < 0.05 (vs. control group; one-way ANOVA), **** *p* < 0.0001 (vs. control group; one-way ANOVA).

*Danio rerio* larvae were xenografted with A375 cells and treated with 7.5 μg/mL of *Lamprocapnos spectabilis* herb extract or 5 μg/mL of a reference drug—etoposide or fish medium E3—as a control (10 animals/group). Figure 5A presents the scheme of the *Danio rerio* larvae xenograft experiments. A moderate, but statistically significant, reduction in the number of cancer cells in *Danio rerio* larvae after their exposure to the investigated extract was observed (Figure 3C and Figure 5B). Inhibition of cancer A375 cell proliferation by *Lamprocapnos spectabilis* herb extract was compared to the activity of the anticancer drug etoposide (Figure 5C).

## 3. Experimental Section

### 3.1. Chemicals and Plant Materials

Acetonitrile (MeCN), methanol (MeOH), and 1-butyl-3-methylimidazolium tetrafluoroborate of chromatographic quality were purchased from E. Merck (Darmstadt, Germany); dimethyl sulfoxide (DMSO) was from Sigma-Aldrich (Saint Louis, MO, USA).

Alkaloid standards (magnoflorine, protopine, stylopine, palmatine, berberine, sanguinarine and chelerythrine) were purchased from Chem Faces Biochemical Co., Ltd. (Wuhan, China), with purity ≥ 98%. Berberine was purchased from Sigma-Aldrich (St. Louis, MO, USA), with purity ≥ 95%.

Plant materials were collected and identified in the Botanical Garden of Maria Curie-Skłodowska University in Lublin (Poland) between April and June 2020. A voucher specimen was deposited in the Department of Inorganic Chemistry, Medical University of Lublin. Plant tissues were separated into herbs and roots and dried at ambient temperature for 2 weeks.

### 3.2. Apparatus and HPLC-DAD Conditions

Analysis was performed using an LC-20AD Shimadzu (Shimadzu Corporation, Canby, OR, USA) liquid chromatograph equipped with Synergi Polar RP 80A (150 mm × 4.6 mm, 5 µm) column. The chromatograph was equipped with a Shimadzu 364 SPD-M20A detector (Shimadzu Corporation, Canby, OR, USA). Detection was carried out at a wavelength of 240 nm. All chromatographic measurements were controlled by a CTO-10ASVP thermostat (Shimadzu Corporation, Canby, OR, USA). The eluent flow rate was 1.0 mL/min, and column temperature 22 °C. Extracts were injected into the column using the Rheodyne 20 µL injector. The DAD detector was set in the 200–800 nm range. Data acquisition and processing were carried out with LabSolutions software (Shimadzu Corporation, Kyoto, Japan). The mobile phase was composed of 0.04 ML^−1^ 1-butyl-3-methylimidazolium tetrafluoroborate in water (solvent A) and 0.04 ML^−1^ 1-butyl-3-methylimidazolium tetrafluoroborate in acetonitrile (solvent B) in gradient elution: 0–20 min, 25% B; 20–30 min, 25–32% B; 30–37 min, 32–40% B, 37–50 min, 60% B, 50–90 min, 60% B. Calibration curves were constructed by analyzing the alkaloid standards at eight concentrations, ranging from 0.01 to 0.2 mg/mL. The calibration curves were obtained by means of the least square method. The limit of detection (LOD) and limit of quantification (LOQ) obtained for the alkaloids were calculated according to the following formulas: LOD = 3.3 (SD/S), and LOQ = 10 (SD/S), where SD is the standard deviation of response (peak area), and S is the slope of the calibration curve. HPLC analyses of alkaloid standards and plant extracts were repeated three times.

### 3.3. LC-MS/MS

Determination of the studied alkaloids was carried out using an LC system equipped with the Agilent XDB-C18 1.8 µm 4.6 × 50 mm column. The column was maintained at 20 °C. The injected sample volume was 10 µL, while the mobile phase was composed of ACN + 0.1% HCOOH (30:70, *v*/*v*) dosed at a flow rate of 0.4 mL/min. The mass spectral analysis was performed on a UHPLC-QTOF/MS model 1260, 6530 Accurate-Mass QTOF LC/MS (Agilent Technologies, Santa Clara, CA, USA) equipped with an ESI interface operating in positive ion mode, with the following set of operational parameters: capillary voltage, 4000 V; nebulizer pressure, 35 psi; drying gas flow, 7 L/min; drying gas temperature, 295 °C; fragmentor 205 V. The parameters of the MS/MS detector were as follows: ion spray voltage, −4500 kV; turbo spray temperature, 450 °C; curtain gas, 20 psi; nebulizing gas, 45 psi; declustering potential, −50 V. The collision energy was set at −30 eVm, and the collision energy spread was 15 eV in the MS/MS experiments. To obtain convincing results, an automated calibration delivery system was used to automatically calibrate MS and MS/MS with every 5 samples in our study. MS and MS/MS mass spectra were recorded across the mass range 40–370 *m*/*z*. The raw data were acquired and quantified with the use of MassHunter Workstation software. Finally, the data were further processed using Microsoft Excel.

### 3.4. Extraction Procedure

The procedure used for extraction was described earlier with small modifications [45,46].

Weighted samples (5 g) of each plant were macerated with 100 mL ethanol for 72 h and continuously extracted in an ultrasonic bath for 5 h. Extracts were filtered, the solvent evaporated under vacuum, and the residues dissolved in 30 mL of 2% sulfuric acid and defatted with diethyl ether (3 × 40 mL). The aqueous layers were subsequently basified with 25% ammonia to a pH of 9.5–10, and the alkaloids extracted with chloroform (3× 50 mL). After evaporation of the organic solvent, the dried extracts were dissolved in 5 mL MeOH prior to HPLC analysis.

In order to determine the % recovery, 5 g of plant materials were sprayed with a 4.2 mL solution of a mixture of standards containing the investigated alkaloids with the following concentrations: sanguinarine and stylopine, 0.03 mg/mL; magnoflorine and chelerythrine, 0.07 mg/mL; protopine, palmatine and berberine, 0.14 mg/mL. After evaporation of the solvent, alkaloids were extracted using the procedure described above.

### 3.5. Investigation of Cytotoxic Activity

#### 3.5.1. Investigation of Cell Viability

Cytotoxicity of the plant extracts was examined against a panel of three melanoma cell lines (A375, G-361, SK-MEL-3) characterized by different degrees of genetic complexity. Fibroblast cells (WS1) were used in order to evaluate the effect of an alkaloid standard (sanguinarine) against normal cells. The investigated cell lines were obtained from American Type Culture Collection (ATCC; Manassas, VA, USA). A375 cells were cultured in Dulbecco’s Modified Eagle’s Medium (DMEM) (Sigma Aldrich, St. Louis, MO, USA) supplemented with 10% heat-inactivated fetal bovine serum (FBS), penicillin (100 U/mL) and streptomycin (100 µg/mL). Human melanoma SK-MEL-3 and G-361 cells were maintained in McCoy’s 5A Medium (Sigma Aldrich, St. Louis, MO, USA) supplemented with 15% (for SK-MEL-3) or 10% (for G-361) FBS, penicillin (100 U/mL) and streptomycin (100 μg/mL). The cells were maintained at 37 °C in a 5% CO2 atmosphere. The dried plant extracts and standard (sanguinarine) were dissolved in DMSO in order to obtain stock solutions at concentrations of 50 mg/mL. On the day of the experiments, the suspension of cells (1 × 105 cells/mL) in respective medium was applied to a 96-well plate at 100 μL per well. After 24 h of incubation, the medium was removed from the wells and replaced by increasing concentrations of plant extracts or standards in medium containing 2% FBS. The control cells were only cultured with a medium containing 2% FBS. Cytotoxicity of DMSO was also evaluated at concentrations present in respective dilutions of stock solutions. After 24 h incubation, 15 μL MTT working solution (5 mg/mL in PBS) was added to each well. The plate was incubated for 3 h. Subsequently, 100 μL of 10% SDS solution was added to each well. Cells were incubated overnight at 37 °C to dissolve the precipitated formazan crystals. The concentration of the dissolved formazan was evaluated by measuring the absorbance at λ = 570 nm using a microplate reader (Epoch, BioTek Instruments, Inc., Winooski, VT, USA). Two independent experiments were performed in triplicate. The results of the MTT assay were expressed as mean ± SD. DMSO in the concentrations present in the dilutions of stock solutions did not influence the viability of the tested cells.

#### 3.5.2. *Danio rerio* Culture and Fish Embryo Toxicity Test (FET)

*Danio rerio* of the AB strain (Experimental Medicine Centre, Medical University of Lublin, Poland) were maintained at 28 ± 0.5 °C under a 14/10 h light/dark cycle with standard aquaculture conditions. After mating, the fertilized eggs were collected within 30 min. Embryos were reared in E3 embryo medium (pH 7.1–7.3; 17.4 µM NaCl, 0.21 µM KCl, 0.12 µM MgSO4 and 0.18 µM Ca(NO_3_)_2_) in an incubator (IN 110 Memmert GmbH, Germany) at 28 ± 0.5 °C. Embryos were examined to remove unfertilized, coagulated and damaged samples. The FET test was performed based on OECD Guideline for the Testing of Chemicals, Test No. 236. Examined extract was weighted, dissolved in DMSO as stock solution, and diluted in E3 embryo medium to the indicated treatment concentrations. Stock and dilutions in E3 embryo medium were freshly prepared before testing. Embryos were exposed to E3 medium (control group) or serial dilutions of the *L. spectabilis* extract (1, 2.5, 5, 7.5, 10, 15, 25, 50 μg/mL). The final DMSO concentration had no detectable effects on zebrafish development. The test was conducted in 24-well plates, 5 embryos per well, 10 per group, in triplicate. The covered plates were kept at 28 ± 0.5 °C under light/dark conditions (12 h/12 h). Embryonic viability and malformation rates of each treatment group were recorded at 24, 48, 72, and 96 hpf. All experiments were conducted in accordance with the National Institute of Health Guidelines for the Care and Use of Laboratory Animals and the European Community Council Directive for the Care and Use of Laboratory Animals (2010/63/EU). For the experiment with larvae up to 5 dpf, approval by the Local Ethical Commission was not required.

#### 3.5.3. *Danio rerio* Human Tumor Cell Xenograft

*Danio rerio* embryos were obtained using standard mating conditions. Before xenotransplantation at 48 hpf, embryos were dechorionized using microforceps, anesthetized with 0.0016% tricaine, and positioned on their left side on a wet Petri dish with microscope slide. A375 cells were detached from culture dishes using 0.25% Trypsin-EDTA and washed twice with PBS at room temperature. Cells were stained with 5 μM DiI diluted in PBS for 20 min at 37 °C and washed three times with PBS (according to manufacturer instructions). Cancer cells were counted by microscopy and injected into the center of the yolk sac using a microinjector (NARISHIGE, IM-300, Japan) with micromanipulator (World Precision Instruments, 3301R, USA) equipped with borosilicate glass capillaries (World Precision Instruments, Sarasota, USA). After injection, embryos were transferred into 96-well plates and incubated in 7.5 μg/mL concentration of the extract, 5 μg/mL of etoposide diluted in E3 media. The control group consisted of injected embryos in E3 medium. Injected embryos were maintained at 32 °C for 3 days and analyzed for cancer cell proliferation.

#### 3.5.4. Quantification of Xenografted Melanoma Cancer Cells

After 3 days post injection (dpi), larvae were anesthetized with 0.0016% tricaine, and the single cell solution was prepared according to the procedure described previously [47]. Fifty microliters of 8% paraformaldehyde solution was used for fixation of the cells. Images were captured with a ZEISS SteREO Discovery, V8 microscope and Zen 2.3 lite software (Carl Zeiss Microscopy GmbH, Jena, Germany). The cells were counted using ImageJ software.

#### 3.5.5. Statistical Analysis

For statistical analysis, GraphPad Prism 5.0 was used (GraphPad Software Inc., La Jolla, CA, USA).

## 4. Conclusions

All tested plant extracts contained isoquinoline alkaloids with cytotoxic activity. Large differences in the content of investigated alkaloids were observed in extracts obtained from various species and different parts of plant.

In most cases, extracts obtained from the roots contained higher concentrations of alkaloids than extracts obtained from herb of the same plant. Only the extract obtained from *Macleaya cordata* leaves contained a higher concentration of isoquinoline alkaloids compared to the extract obtained from the roots.

Almost all investigated plant extracts exhibited high cytotoxic activity against all tested cancer cell lines, especially against A375 and G-361 cells. Extracts from *Macleaya cordata* leaves, stalk, and root and *Lamprocapnos spectabilis* herb exhibited higher cytotoxic activity against all tested melanoma cell lines.

The differences in cytotoxicity of extracts obtained from various parts of the investigated plants were strongly dependent on the content of isoquinoline alkaloids.

In vivo experiments, for the first time using the *Danio rerio* larvae xenograft model for the determination of cytotoxicity of *Lamprocapnos spectabilis* herb extract, confirmed a significant effect of the extract on the decrease in melanoma cell numbers in a living organism.

The Investigated extracts, especially those obtained from *Macleaya cordata* and *Lamprocapnos spectabilis* herb, may be recommended for further investigations as new candidates for anticancer agents.

## Figures and Tables

**Table 1:** Values of retention time (t_R_), asymmetry factor (A_S_), and theoretical plate number per meter (N/m) obtained for alkaloid standards.

Name of Compound	t_R_	A_S_	N/m
Magnoflorine	5.34	1.02	21,900
Protopine	14.18	1.01	54,300
Stylopine	19.73	0.99	61,100
Palmatine	30.56	1.10	171,100
Berberine	35.48	1.01	342,400
Sanguinarine	36.82	0.99	490,200
Chelerythrine	42.57	1.11	90,600

**Table 2:** MS parameters used for determination and identification of selected alkaloids in plant extract samples.

Compound	Elemental Composition	Polarity	Theoretical (*m*/*z*)	Measured (*m*/*z*)	Major Fragment Ions	Error (ppm)	ID Score [%]
Magnoflorine	C_20_H_24_NO_4_ [M + H]^+^	ESI+	341.7915	341.7917	296.7147 264.6899 236.7089 206.7327	−0.39	99.48
Protopine	C_20_H_20_NO_5_ [M + H]^+^	ESI+	353.7653	353.7655	336.1209 274.6716 205.7380 188.7681 148.8711	−0.93	99.15
Stylopine	C_19_H_17_NO_4_ [M + H]^+^	ESI+	331.7109	331.7105	277.8803 251.7101 175.7931 163.8332 148.8696	−0.48	99.63
Palmatine	C_21_H_22_NO_4_ [M + H]^+^	ESI+	351.7872	351.7853	335.7442 307.7351 277.6835 249.6977	1.07	99.62
Berberine	C_20_H_18_NO_4_ [M + H]^+^	ESI+	335.7426	335.7429	319.7029 305.6823 304.1893 291.6987 277.6827	−0.41	99.18
Sanguinarine	C_20_H_14_NO_4_ [M + H]^+^	ESI+	331.7068	331.7065	316.6761 303.6993 288.6723 273.6853 245.7012	1.26	99.34
Chelerythrine	C_21_H_18_NO_4_ [M + H]^+^	ESI+	347.7491	347.7489	331.7071 303.6990 274.6920 231.6968	1.19	99.56

**Table 3:** Equation of calibration curve, correlation coefficients (r), limit of detection (LOD) and limit of quantification (LOQ) values.

Alkaloid	Equation of Calibration Curve	r	LOD [mg/mL]	LOQ [mg/mL]
Magnoflorine	y = 25635805 x − 251782	0.9983	0.0222	0.0672
Protopine	y = 25340599 x + 98298	0.9935	0.0222	0.0673
Stylopine	y = 879342 x − 13994	0.9964	0.0241	0.0729
Palmatine	y = 52900121 x + 732112	0.9945	0.0202	0.0613
Berberine	y = 70984852 x − 330076	0.9979	0.0133	0.0403
Sanguinarine	y = 63958474 x + 118255	0.9995	0.0060	0.0182
Chelerythrine	y = 57154059 x + 12978	0.9990	0.0086	0.0260

**Table 4:** Content of alkaloids in plant samples.

Name of Compound	Content of Alkaloids (mg/g of Dry Plant Material) and Standard Deviation of These Values.											
	*Macleaya cordata* Leaves	*Macleaya cordata* Stalk	*Macleaya cordata* Root	*Pseudo-fumaria lutea* Herb	*Pseudo-fumaria lutea* Root	*Lamprocapnos spectabilis* Herb	*Lamprocapnos Spectabilis* Root	*Fumaria* *officinalis*	*Meconopsis cambrica* Herb	*Meconopsis cambrica* Root	*Thalictrum foetidum* Herb	*Thalictrum foetidum* Root
Magnoflorine	ND	ND	ND	ND	ND	ND	ND	ND	ND	ND	ND	0.021 ± 0.002
Protopine	0.015 ± 0.0011	0.010 ± 0.001	0.018 ± 0.0015	0.120 ± 0.011	0.370 ± 0.031	0.448 ± 0.039	3.350 ± 0.28	0.514 ± 0.047	0.093 ± 0.008	0.700± 0.0067	ND	ND
Stylopine	ND	ND	ND	0.655 ± 0.064	5.716 ± 0.495	ND	ND	ND	ND	ND	ND	ND
Palmatine	ND	ND	ND	0.100 ± 0.008	0.268 ± 0.022	ND	ND	ND	ND	ND	ND	ND
Berberine	ND	ND	ND	ND	ND	ND	ND	ND	ND	ND	0.001 ± 0.0001	0.308 ± 0.028
Sanguinarine	0.051 ± 0.003	0.027 ± 0.002	0.026 ± 0.002	ND	ND	0.066 ± 0.006	0.097 ± 0.008	0.043 ± 0.004	0.005 ± 0.004	0.047 ± 0.003	ND	ND
Chelerythrine	0.046 ± 0.003	0.024 ± 0.002	0.019 ± 0.002	ND	ND	ND	ND	ND	ND	ND	ND	ND

ND, not detected.

**Table 5:** Cytotoxic activity expressed as IC_50_ values of the investigated extracts against melanoma cell lines (A375, G-361, SK-MEL-3).

IC_50_ [µg/mL] for Cell Viability			
	A375	G-361	SK-MEL-3
*Macleaya cordata* leaves	0.75 (±0.04)	1.51 (±0.01)	0.21 (±0.02)
*Macleaya cordata* stalk	0.77 (±0.02)	1.12 (±0.01)	0.14 (±0.01)
*Macleaya cordata* root	7.13 (±0.31)	5.38 (±0.27)	2.44 (±0.32)
*Pseudofumaria lutea* herb	21.57 (±1.36)	7.96 (±0.35)	46.67 (±4.11)
*Pseudofumaria lutea* root	110.29 (±4.07)	6.63 (±0.41)	40.66 (±2.79)
*Lamprocapnos spectabilis* herb	4.13 (±0.11)	11.45 (±0.55)	23.22 (±3.01)
*Lamprocapnos spectabilis* root	extract insoluble in DMSO and H_2_O—analysis not performed		
*Fumaria officinalis* whole plant	141.9 (±5.82)	11.79 (±1.01)	124.49 (±7.81)
*Meconopsis cambrica* herb	22.53 (±0.93)	11.72 (±0.97)	88.54 (±5.74)
*Meconopsis cambrica* root	40.13 (±1.38)	70.60 (±6.25)	94.10 (±6.07)
*Thalictrum foetidum* herb	64.78 (±2.55)	9.72 (±1.10)	43.90 (±4.01)
*Thalictrum foetidum* root	37.01 (±2.13)	6.69 (±0.22)	15.96 (±0.39)

## Data Availability

Not applicable.

## Supplementary Materials

The following supporting information can be downloaded at: https://www.mdpi.com/article/10.3390/molecules28083503/s1, Figure S1: Representative MS (A) and MS/MS (B) spectra for the studied alkaloids obtained from Lamprocapnos spectabilis herb.
