# Supplementary material for: Determination of Some Isoquinoline Alkaloids in Extracts Obtained from Selected Plants of the Ranunculaceae, Papaveraceae and Fumarioideae Families by Liquid Chromatography and In Vitro and In Vivo Investigations of Their Cytotoxic Activity
Source: Molecules. 2023 Apr 16;28(8):3503. doi: 10.3390/molecules28083503 (PMC10143472; doi:10.3390/molecules28083503)
Supplement: Supplementary file 1 [file molecules-28-03503-s001.zip › molecules-2325084-supplementary.pdf]

described previously [20,42].

(A)

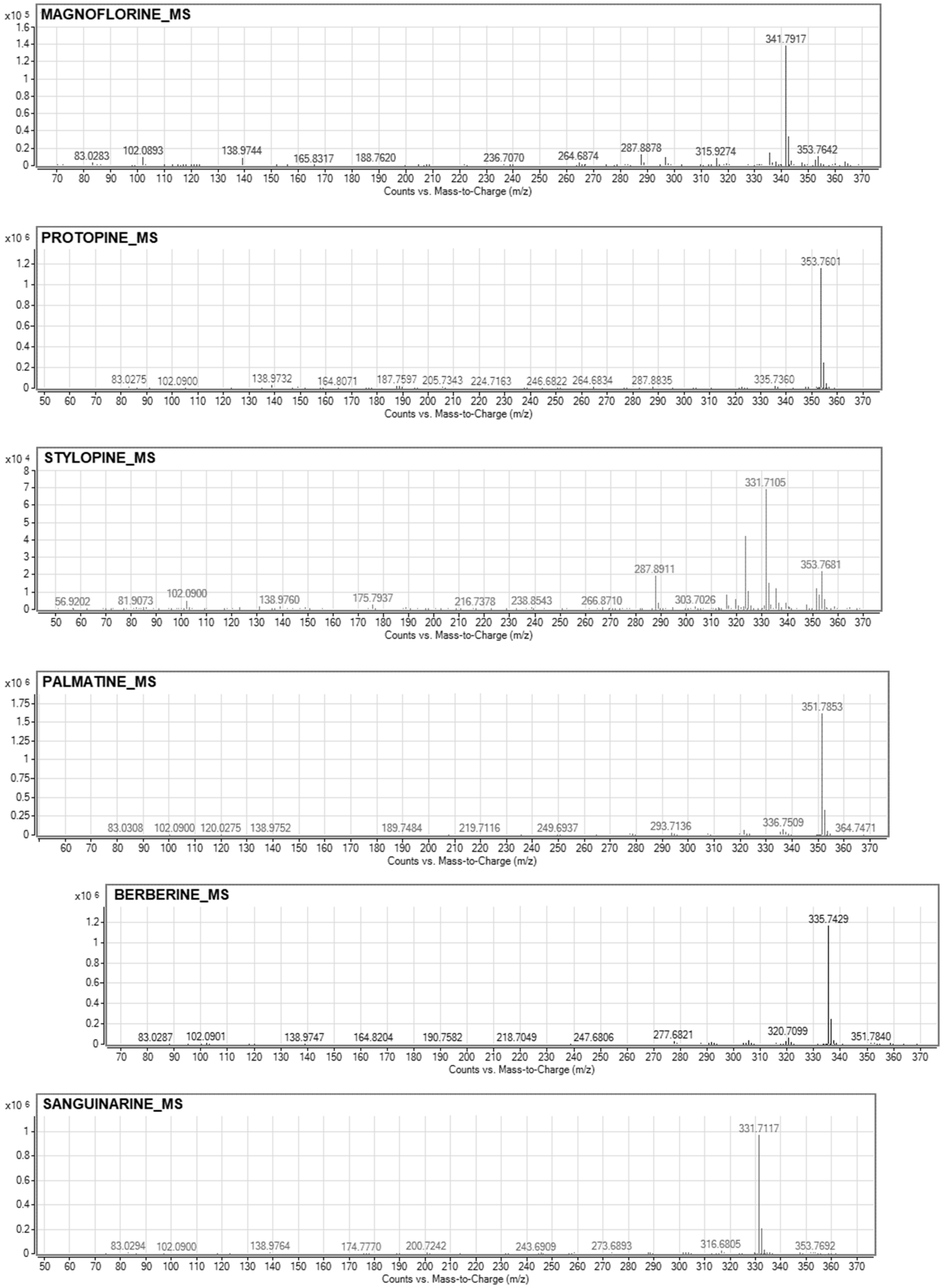

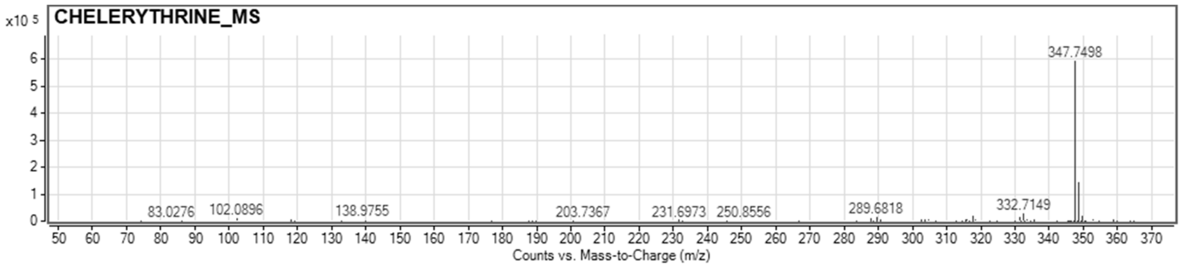
CHELERYTHRINE_MS
x10 5
347.7498
83.0276
102.0896
138.9755
203.7367
231.6973
250.8556
289.6818
332.7149
Counts vs. Mass-to-Charge (m/z)

(B)

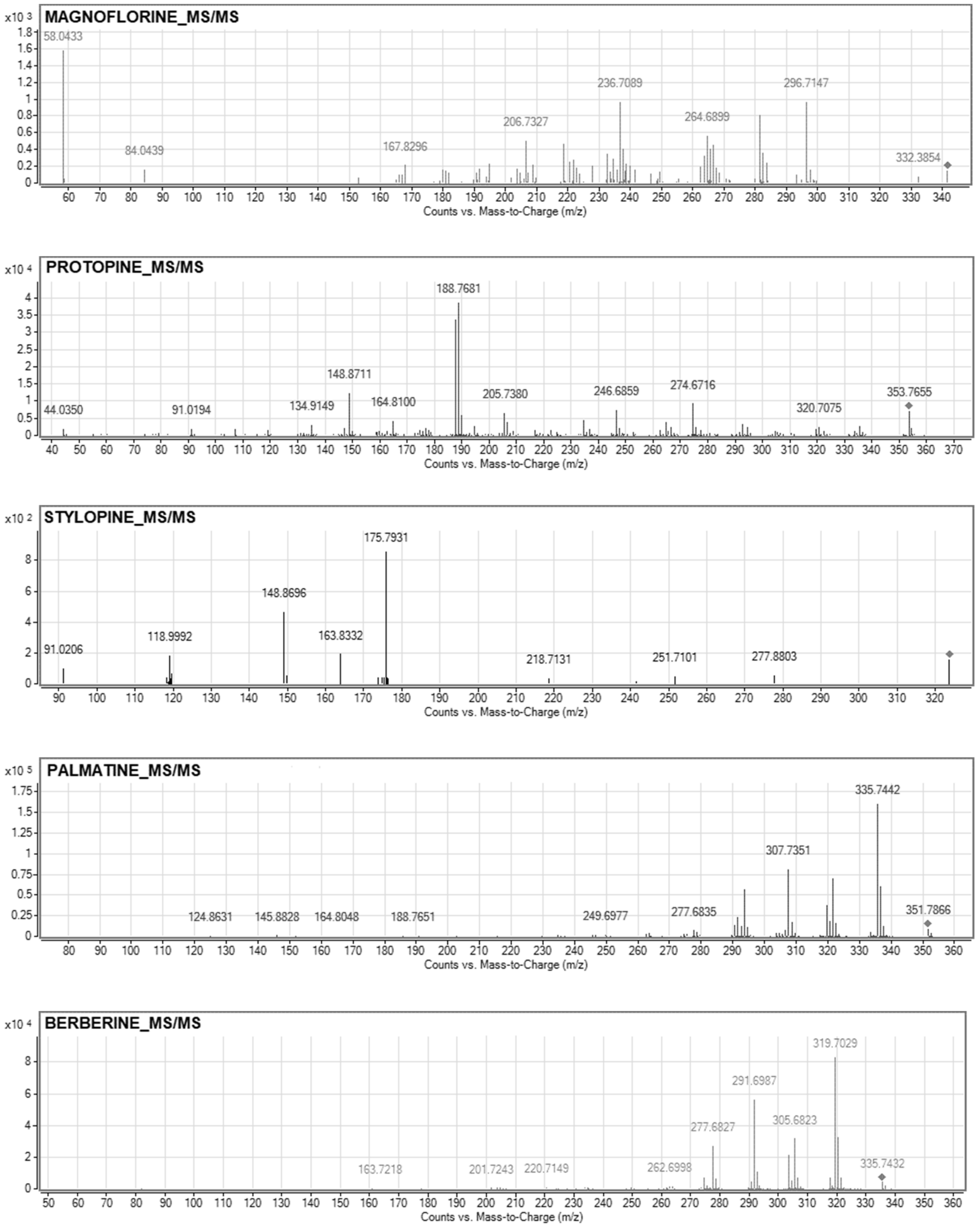
MAGNOFLORINE_MS/MS
x10 3
58.0433
84.0439
167.8296
206.7327
236.7089
264.6899
296.7147
332.3854
Counts vs. Mass-to-Charge (m/z)
PROTOPINE_MS/MS
x10 4
44.0350
91.0194
134.9149
148.8711
164.8100
188.7681
205.7380
246.6859
274.6716
320.7075
353.7655
Counts vs. Mass-to-Charge (m/z)
STYLOPINE_MS/MS
x10 2
91.0206
118.9992
148.8696
163.8332
175.7931
218.7131
251.7101
277.8803
Counts vs. Mass-to-Charge (m/z)
PALMATINE_MS/MS
x10 5
124.8631
145.8828
164.8048
188.7651
249.6977
277.6835
307.7351
335.7442
351.7866
Counts vs. Mass-to-Charge (m/z)
BERBERINE_MS/MS
x10 4
163.7218
201.7243
220.7149
262.6998
277.6827
291.6987
305.6823
319.7029
335.7432
Counts vs. Mass-to-Charge (m/z)

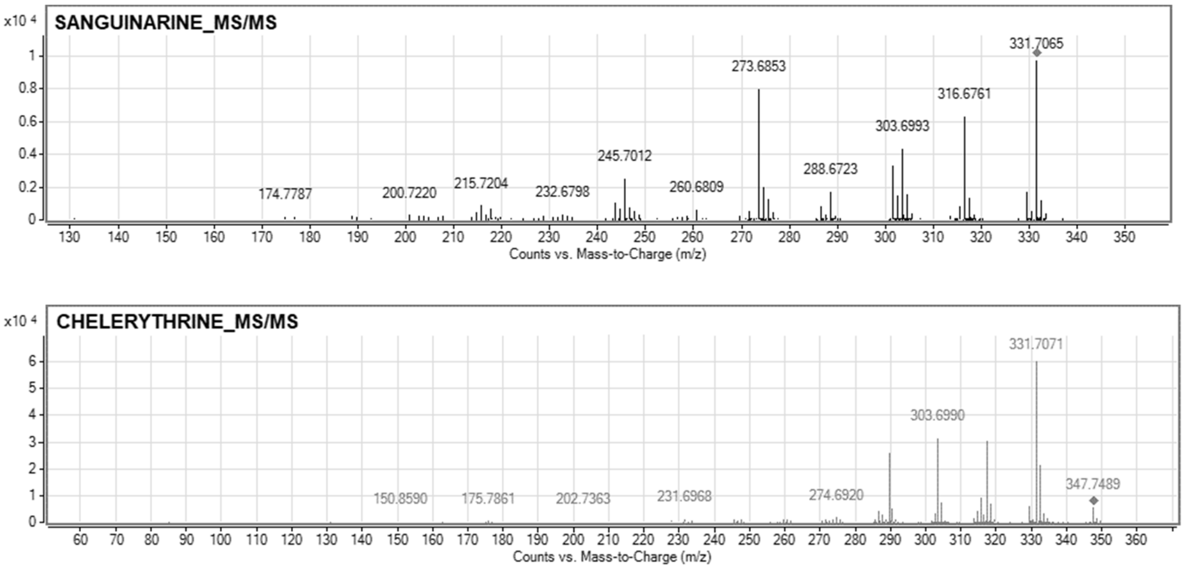

Figure S1. Representative MS (A) and MS/MS (B) spectra for the studied alkaloids obtained from *Lamprocapnos spectabilis* herb.
